# Use of antibiotics by primary care doctors in Hong Kong

**DOI:** 10.1186/1447-056X-8-5

**Published:** 2009-05-22

**Authors:** Tai Pong Lam, Pak Leung Ho, Kwok Fai Lam, Kin Choi, Raymond Yung

**Affiliations:** 1 Family Medicine Unit, Department of Medicine, The University of Hong Kong, Hong Kong; 2 Department of Microbiology, The University of Hong Kong, Hong Kong; 3 Department of Statistics and Actuarial Science, The University of Hong Kong, Hong Kong; 4 Hong Kong Medical Association, Hong Kong; 5 Hong Kong Sanatorium & Hospital, Hong Kong

## Abstract

**Objectives:**

To determine the use of antibiotics by primary care doctors.

**Methods:**

General practitioners in Hong Kong were invited to fill in a short questionnaire on every patient with infection that they had seen on the first full working day once every three months for four consecutive quarters starting from December 2005.

**Results:**

Forty six primary care doctors took part and a total of 3096 completed questionnaires were returned. The top three diagnoses were upper respiratory tract infection (46.7%), gastrointestinal infection (8.2%) and pharyngitis (7.1%). Thirty percent of patient encounters with infections were prescribed antibiotics but only 5.2% of patient encounters with upper respiratory tract infection (URTI) were prescribed antibiotics. Amino-penicillins were the most commonly used antibiotics while beta-lactam/beta-lactamase inhibitor combinations (BLBLIs) were the second most commonly used antibiotics and they accounted for 16.5% and 14.0% of all antibiotics used respectively. Of all patients or their carers, those who demanded or wished for antibiotics were far more likely to be prescribed antibiotics (Pearson chi-square test, p < 0.0001). Those patients who were attending the doctors for follow-up consultations were also more likely to be prescribed antibiotics (Pearson chi-square test, p < 0.001).

**Conclusion:**

The antibiotic prescribing patterns of primary care doctors in Hong Kong are broadly similar to primary care doctors in other developed countries but a relatively low rate of antibiotics is used for URTI.

## Introduction

Overuse of antibiotics is a worldwide phenomenon [1,2] and it contributes to the emergence of antimicrobial resistance [3-5]. Unnecessary use of antibiotics also leads to an increased risk of side effects [6], increased medical care costs [7] and medicalising effects [8].

In the Fifty-eighth World Health Assembly held in May 2005, it was resolved and agreed by more than 60 countries that the containment of antimicrobial resistance is an international goal; however the strategy for doing this has not been widely implemented. This assembly urged member states to (a) enhance the rational use of antimicrobial agents by developing and enforcing national standard practice guidelines for common infections, in both the public and private health sectors, (b) monitor the use of antimicrobial agents regularly, as well as the level of antimicrobial resistance in all relevant sectors, and (c) actively share knowledge and experience about best practices in promoting the rational use of antimicrobial agents.

Most antibiotics are prescribed by primary care doctors. Many of these are for infections of the respiratory tract [9] despite research studies demonstrating little or no clinical benefits [10-17]. Studies done by Lam and Lam revealed that in Hong Kong antibiotics are frequently used for patients with respiratory tract infections [18]. Many doctors in Hong Kong have also acknowledged that they might be prescribing antibiotics too often for upper respiratory tract infections (URTI) [19]. Possible reasons for this could involve misconceptions about the significance of fever, discoloured sputum or nasal discharge; the presence of tonsillar exudates and/or cervical lymphadenopathy [18] as indicators of bacterial infections. Patients' expectations of receiving antibiotics were also cited as a major reason for prescribing them [19]. These findings were however based on the primary care doctors' report of their clinical behaviours, which may have resulted in an underestimate, or even an overestimate of their use of antibiotics. At present no other current information is available about the actual usage of antibiotics by primary care doctors in Hong Kong.

This study thus represents a step forward in the understanding of the use of antibiotics by primary care doctors in Hong Kong. It aims to examine the primary care doctors' clinical behaviour in the use of antibiotics by detailing the type of antibiotics they use and the illnesses that they use them for. A similar study in Scandinavia demonstrated benefit in reducing antibiotic use in that country [20].

## Subjects and methods

Hong Kong's health care delivery system is structured around general family practice, with specialist support available both privately and through public hospitals. Western-trained private medical practitioners provide 75% of primary care while public doctors provide 15% and the rest by other health care providers, such as Traditional Chinese Medicine practitioners. In 2005, there were about 10500 registered doctors. It was estimated that more than 5000 doctors worked in the public hospitals and clinics, and 5000 in the community (including specialists and primary care doctors in both groups). Most of the others probably spent most of their time outside Hong Kong but continued to be registered. There was no separate list of family doctors.

All registered medical practitioners in Hong Kong were sent invitation letters to participate in the research study in October 2005 and 46 primary care doctors agreed to participate.

Participants were requested to fill in a short instrument on every patient with infection that they had seen on the first full working day, once every three months for four consecutive quarters starting from December 2005 (i.e. 1 December 2005; 1 March 2006; 1 June 2006; and 1 September 2006). The instrument consisted of 9 items (Appendix A [see Additional file 1]). Items 1 and 2 gave the demographical characteristics including gender and age of patient, whereas items 3 and 9 specified the nature of the consultation (acute vs. scheduled; the first vs. a later one). In item 4, respondents were asked to select only the most important one from a range of infections as the main diagnosis, and space was provided for them to specify other infections not listed. Item 6 represented respondent's best interpretation of patient's expectations for antibiotics whereas item 5 asked if an antibiotic was prescribed and item 7 requested details of the prescribed antibiotic drug (including name, frequency, dosage, and duration). Item 8 explored the factors influencing the choice of antibiotic drugs (multiple answers were allowed). All items needed to be completed for every patient prescribed antibiotics. For cases where antibiotics were not prescribed, all except items 7 and 8 should be filled in. The questionnaire was anonymous, yet participants were encouraged to put down their internal reference of patient code number in the space provided for the sake of traceability. Participants were also asked to complete a separate form on some of their personal particulars (including age, gender, years of clinical practice after graduation, type of practice, and qualifications).

Data were analyzed using JMP for Windows (Release 6.0.2). Pearson chi-square test was performed to test for the existence of any association between two ordinal or nominal variables. A p-value < 0.05 was considered statistically significant.

## Results

By September 2006, 3096 completed questionnaires had been returned by 46 participating primary care doctors. Thirty five doctors returned questionnaires on all the four days and the remaining 11 doctors participated for 1 to 3 days. There were 37 males and 9 females. Their ages ranged from 28–70 years (median 48). They had been in clinical practice for between 2 and 44 years (median 19.5). There were more primary care doctors working in the private than the public sector (95.6% vs. 4.4%). 58.7% of the participants had higher qualifications in Family Medicine/General Practice. Twenty nine (63.0%) participants obtained their primary medical qualification from Hong Kong, 7 (15.2%) from Europe (including UK and Ireland), 4 (8.7%) from Australasia, 4 (8.7%) from Mainland China or Taiwan and the rest from other parts of the world.

The joint distribution of age and sex of the patient encounters reported is shown in Table 1. Of all patient encounters, 58.4% were for female patients and 85.5% were for acute consultations. The distribution of the ten commonest diagnoses is shown in Table 2 and the top three were URTI (46.7%), gastrointestinal infection (8.2%), and pharyngitis (7.1%). Thirty percent of patients with infections were prescribed antibiotic therapy (95% CI 0.285–0.317).

**Table 1:** Age distribution of patients reported

Age	Females (%)	Males (%)
0–5	131 (4.2%)	138 (4.5%)

6–14	129 (4.2%)	135 (4.4%)

15–20	80 (2.6%)	63 (2.0%)

21–30	366 (11.8%)	182 (5.9%)

31–40	419 (13.5%)	216 (7.0%)

41–50	276 (8.9%)	202 (6.5%)

51–60	153 (4.9%)	148 (4.8%)

61–70	105 (3.4%)	84 (2.7%)

Over 70	144 (4.7%)	93 (3.0%)

Missing	32 (1.0%)	

**Table 2:** Top 10 diagnoses made by the primary care doctors

Diagnosis	Number of patients not prescribed antibiotics (%)	Number of patients prescribed antibiotics (%)
Unspecified upper respiratory tract infection (URTI)	1372 (94.8%)	75 (5.2%)

Gastrointestinal infection	241 (94.5%)	14 (5.5%)

Pharyngitis	106 (48.0%)	115 (52.0%)

Acute bronchitis	70 (38.3%)	113 (61.8%)

Skin and soft tissue infection (including erysipelas, cellulitis, abscess, and wound infection)	34 (18.8%)	147 (81.2%)

Urinary tract infection	9 (8.7%)	94 (91.3%)

Tonsillitis	14 (20.3%)	55 (79.7%)

Conjunctivitis	6 (9.2%)	59 (90.8%)

Acute infection exacerbating a chronic pulmonary disease	11 (26.2%)	31 (73.8%)

Sinusitis	9 (23.1%)	30 (76.9%)

Of all patients or their carers, three quarters had no expectation of antibiotics by the doctors wherever such an assessment was possible (Table 3). However, those who demanded or wished for antibiotics (17.9% of all patients) were far more likely to be prescribed antibiotics (Pearson chi-square test, p < 0.0001).

**Table 3:** Patients' or carers' expectation of antibiotics

Expectation	Patients' or carers' expectation of antibiotics in number (% of total)	Number of patients not prescribed antibiotics (%)	Number of patients prescribed antibiotics (%)
Demanded	71(2.5%)	5 (7.0%)	66 (93.0%)

Wished for	432 (15.4%)	72 (16.7%)	360 (83.3%)

Neutral	2106 (75.1%)	1622 (77.0%)	484 (23.0%)

Reluctant	182 (6.5%)	175 (96.2%)	7 (3.8%)

Resisted	12 (0.4%)	12 (100.0%)	0 (0.0%)

Table 4 shows the 10 most frequently prescribed antibiotics by primary care doctors. Amino-penicillins and beta-lactam/beta-lactamase inhibitor combinations (BLBLIs) were by far the two most commonly prescribed antibiotics and they accounted for 16.5% and 14.0% of all antibiotics used respectively. A relatively new antibiotic, azithromycin, accounted for 3.9% of all antibiotics used.

**Table 4:** Top 10 antibiotics prescribed by the primary care doctors

Antibiotics*	Number of patients prescribed the antibiotics	Percentage of patients prescribed the antibiotics
Aminopenicillins†	153	16.5%

BLBLIs‡	130	14.0%

Clarithromycin	68	7.3%

Chloramphenicol	57	6.2%

Cefuroxime	40	4.3%

Metronidazole	39	4.2%

Erthromycin	37	4.0%

Levofloxacin	37	4.0%

Azithromycin	36	3.9%

Ampicillin plus cloxacillin	35	3.8%

* These ten antibiotics together account for 68.2% of the total antibiotic use

† Including ampicillin and amoxicillin

‡ BLBLI, beta-lactam and beta-lactamase inhibitor combinations including amoxicillin-clavulanate and ampicillin-sulbactam

It was also found that 47.2% of patients who attended follow up consultations for their infective illnesses were prescribed antibiotics vs. 26.3% of those who attended for first consultation for the episode of infective illness (Pearson chi-square test, p < 0.001).

## Discussion

This study aimed to examine the use, and associated determinants of, antibiotic prescribing by primary care doctors in Hong Kong. The findings of this study were able to reveal that while the antibiotic prescribing behaviour of primary care doctors in Hong Kong is broadly similar to that of developed countries, however, certain aspects of their antibiotic prescribing are different from those of other countries. For instance, the antibiotic prescribing rate for patients with URTI was only 5.2% and this was significantly lower than the 44% reported by Petersen & Hayward [21] on antibiotics used by general practitioners in the UK. Furthermore, a relatively new antibiotic, azithromycin, was the ninth most commonly used antibiotic in this study, and accounted for 3.9% of total antibiotic used but it was outside of the top 20 antibiotics used in Petersen & Hayward's study. This showed that some primary care doctors in Hong Kong are more ready to use newer generations of antibiotics than their UK counterparts. This pattern of antibiotic use is worthy of special attention from the health authority and may have contributed to put Hong Kong being in the forefront of antibiotic resistance [22].

It is also interesting to note the close relationship between patients' or carers' expectation of antibiotics and the issue of "nonpharmacological" prescriptions. This confirms the previous findings by Lam and Lam [19] and highlights the importance of factors, other than medical, for the inappropriate use of antibiotics worldwide [23].

The fact that patients who were being seen again for the same episode of infective illness were more likely to be prescribed antibiotics could be due to the more serious nature of the infections and/or doctors' concern about the deterioration of patients' conditions. However, it could also represent the strategy adopted by many primary care doctors in reducing antibiotic use when they specifically asked patients to return only if their conditions did not improve.

It is interesting to note that the top 5 conditions that antibiotics were most frequently prescribed for in our study were, in descending order, urinary tract infection (91.3%), conjunctivitis (90.8%), skin and soft tissue infection (81.2%), tonsillitis (79.7%) and sinusitis (76.9%). The results are quite similar to a large study done in the UK [21] where their findings were impetigo (90.7%), conjunctivitis (85.4%), sinusitis (84.9%), urinary tract infection (83.4%), lower respiratory tract infection (82.4%).

## Limitations

The participating doctors of this study belong to a volunteer group of primary care physicians in Hong Kong. Their reported prescribing behaviour may be different from their usual practice and hence this may have affected the accuracy of our findings. However, it was emphasized to the participants that we would like their prescribing behaviour on the reporting days to be the same as other days. There are no reasons to suspect that they might have reported more socially desirable prescribing behaviour as their participation was entirely voluntary. Since Hong Kong, like most countries in Asia, does not have a government funded pharmaceutical scheme, studies of this kind would have to depend on the reporting behaviour of the doctors unless direct access to patients' medical records is allowed. The fact that over 3000 questionnaires were completed for this study would make it the biggest of its kind in Hong Kong thus far.

## Conclusion

The antibiotic prescribing behaviour of primary care doctors in Hong Kong is similar to that of the developed countries but a relatively low rate of antibiotics is used for URTI.

## Competing interests

The authors declare that they have no competing interests.

## Authors' contributions

TPL was the principal investigator of the study and involved in designing the study, supervising the data collection, reviewing/analyzing the data and writing the paper. PLH was involved in conceiving/initiating and designing the study, and writing the paper. KFL was involved in designing the study and did the statistical analyses and contributed to the interpretation of data and writing the paper. KC and RY was involved in designing the study and writing the paper. All authors read and approved the final manuscript.

## Supplementary Material

Additional file 1 **Appendix A – Instrument for the study on use of antibiotics by primary care doctors in Hong Kong**. The sample instrument sent to the participating doctors in the study Click here for file
