# Supplementary material for: Use of antibiotics by primary care doctors in Hong Kong
Source: Asia Pac Fam Med. 2009 May 22;8(1):5. doi: 10.1186/1447-056X-8-5 (PMC2692843; doi:10.1186/1447-056X-8-5)
Supplement: Additional file 1 — Appendix A – Instrument for the study on use of antibiotics by primary care doctors in Hong Kong. The sample instrument sent to the participating doctors in the study [file 1447-056X-8-5-S1.doc]

**Appendix A**

**Use of antibiotics by primary care doctors in Hong Kong**

patient code number ________________

*Please tick the appropriate box.*

1. Age group of patient

| 1 | 0 – 5 |  | 4 | 21 – 30 |  | 7 | 51 – 60 |
| --- | --- | --- | --- | --- | --- | --- | --- |
|  |  |  |  |  |  |  |  |
| 2 | 6 – 14 |  | 5 | 31 – 40 |  | 8 | 61 – 70 |
|  |  |  |  |  |  |  |  |
| 3 | 15 – 20 |  | 6 | 41 – 50 |  | 9 | Over 70 |

1. Gender

| 1 | female |  | 2 | male |
| --- | --- | --- | --- | --- |

1. The consultation is...

| 1 | an acute consultation |  |  | 2 | a scheduled consultation |
| --- | --- | --- | --- | --- | --- |

1. Main diagnosis (only the most important one)

| 1 | otitis media |  | 10 | skin and soft tissue infection (including erysipelas, cellulitis, abscess, and wound infection) |
| --- | --- | --- | --- | --- |
|  |  |
|  |  |  |  |  |
| 2 | tonsillitis |  | 11 | breast infection (including mastitis and breast abscess) |
|  |  |
|  |  |  |  |  |
| 3 | sinusitis |  | 12 | urinary tract infection |
|  |  |  |  |  |
| 4 | pharyngitis |  | 13 | helicobacter infection |
|  |  |  |  |  |
| 5 | unspecified upper respiratory tract infection (URI) |  | 14 | gastrointestinal infection |
|  |  |
|  |  |  |  |  |
| 6 | acute bronchitis |  | 15 | sexually transmitted disease (STD) |
|  |  |  |  |  |
| 7 | acute infection exacerbating a chronic pulmonary disease |  | 16 | gynaecological infection |
|  |  |
|  |  |  |  |  |
| 8 | pneumonia |  | 17 | other infection - please specify: |
|  |  |  |  |  |
| 9 | conjunctivitis |  |  | ________________________________ |

1. Did you prescribe an antibiotic to the patient?

(If you make the decision later, i.e. after the arrival of lab results, please complete this paper then)

| 1 | no |  | 2 | yes |
| --- | --- | --- | --- | --- |

1. What kind of expectations did the patient have on antibiotic prescribing?

(Please tick the box that in your opinion best illustrates the patient’s expectations)

| demanded | | |  | wished for | | | |  | neutral | | | |  | reluctant | | | |  | resisted | | |
| --- | --- | --- | --- | --- | --- | --- | --- | --- | --- | --- | --- | --- | --- | --- | --- | --- | --- | --- | --- | --- | --- |
|  |  |  | | |  | |  | | |  | |  | | |  | |  | | |  |  |
|  | 1 |  | | | 2 |  | | | | | 3 |  | | | | 4 |  | | | 5 |  |

1. Prescribed antibiotic drug(s):

| name of antibiotic drug |  | frequency  (e.g. tds) |  | dosage  (e.g. mg) |  | duration  (days) |
| --- | --- | --- | --- | --- | --- | --- |
|  |  |  |  |  |  |  |
|  |  |  |  |  |  |  |
| name of antibiotic drug |  | frequency  (e.g. tds) |  | dosage  (e.g. mg) |  | duration  (days) |

1. Did any of the following factors influence the choice of antibiotic drug(s) mentioned in item 7 above? (You may tick more than one box)

| 1 | (suspected) allergy |  | 4 | patient’s demand |
| --- | --- | --- | --- | --- |
|  |  |  |  |  |
| 2 | previous side-effect |  | 5 | none of the above |
|  |  |  |  |  |
| 3 | pregnancy/breast feeding |  |  |  |

1. This consultation was...

| 1 | the first one for this episode of illness |
| --- | --- |
|  |  |
| 2 | a later one. No. of consultations all together: ________________ |
